# Gross appearance of the fetal membrane on the placental surface is associated with histological chorioamnionitis and neonatal respiratory disorders

**DOI:** 10.1371/journal.pone.0242579

**Published:** 2020-11-30

**Authors:** Yoshimasa Horikoshi, Chizuko Yaguchi, Naomi Furuta-Isomura, Toshiya Itoh, Kenta Kawai, Tomoaki Oda, Masako Matsumoto, Yukiko Kohmura-Kobayashi, Naoaki Tamura, Toshiyuki Uchida, Naohiro Kanayama, Hiroaki Itoh

**Affiliations:** Department of Obstetrics and Gynecology, Hamamatsu University School of Medicine, Hamamatsu, Japan; BC Children's Hospital, CANADA

## Abstract

An opaque fetal membrane based on gross appearance is traditionally indicative of histological chorioamnionitis; however, to the best of our knowledge, there is currently no supportive evidence, and its diagnostic efficiency has not yet been scientifically demonstrated. The present study aimed to provide scientific insights into the traditional concept of an opaque fetal membrane based on gross appearance being an indicator of histological chorioamnionitis. We examined the placental pathology after screening of the placental gross appearance and perinatal complications and did not examine uncomplicated deliveries. We investigated the relationship between the presence of an opaque fetal membrane and histological chorioamnionitis (Cohort 1, 571 placentas) or the outcomes of neonates delivered at term (Cohort 2, 409 placentas) at Hamamatsu University School of Medicine between 2010 and 2017. The judgment of a positive opaque fetal membrane based on gross appearance correlated with histological chorioamnionitis (Cohort 1). Its sensitivity and specificity were 66.7 and 89.9%, respectively, while positive and negative predictive values were 86.8 and 73.0%, respectively. The judgment of a positive opaque fetal membrane based on gross appearance significantly correlated with chorioamnionitis-related complications in term newborns after adjustments for confounding factors (OR;1.82 [1.07–3.11], P<0.05) (Cohort 2). A correlation was observed even after adjustments for confounding factors. The present study is the first to demonstrate that the judgment of a positive opaque fetal membrane based on gross appearance correlated with histological chorioamnionitis as well as chorioamnionitis-related complications in newborns delivered at term. The present results provide support for the traditionally-described importance of gross inspections for an opaque fetal membrane soon after birth.

## Introduction

Intrauterine infection is associated with various maternal and neonatal adverse outcomes, such as preterm delivery and neonatal respiratory disorders [1–6]. Fifty years ago, Blanc described the histological concept of chorioamnionitis as the infiltration of neutrophils in fetal membranes (amnion and chorion, but not decidua) due to bacterial infection [7, 8]. The relationship between histological chorioamnionitis and neonatal outcomes was supported by the findings of retrospective cohort studies [3, 6]. However, the pathological diagnosis of chorioamnionitis has not contributed to better neonatal outcomes because pathological examinations are generally conducted several days after delivery and early immediate care is critical for neonatal disorders associated with intrauterine infections. Nevertheless, an opaque fetal membrane based on gross appearance is traditionally indicative of histological chorioamnionitis [9–14]; however, to the best of our knowledge, there is currently no supportive evidence and its diagnostic efficiency has not yet been scientifically demonstrated. Furthermore, it is unclear whether the acknowledgment of an opaque fetal membrane based on gross appearance predicts the adverse neonatal outcomes associated with intrauterine infections.

Therefore, the present study aimed to provide scientific insights into the traditional concept of an opaque fetal membrane as an indicator of histological chorioamnionitis. The specific objectives of the present study were to examine the relationship between the presence of an opaque fetal membrane based on gross appearance and histological chorioamnionitis (Cohort 1, 571 placentas) or the outcomes of neonates delivered at term (Cohort 2, 409 placentas) at Hamamatsu University School of Medicine between 2010 and 2017.

## Materials and methods

### Subjects

**Fig 1:**
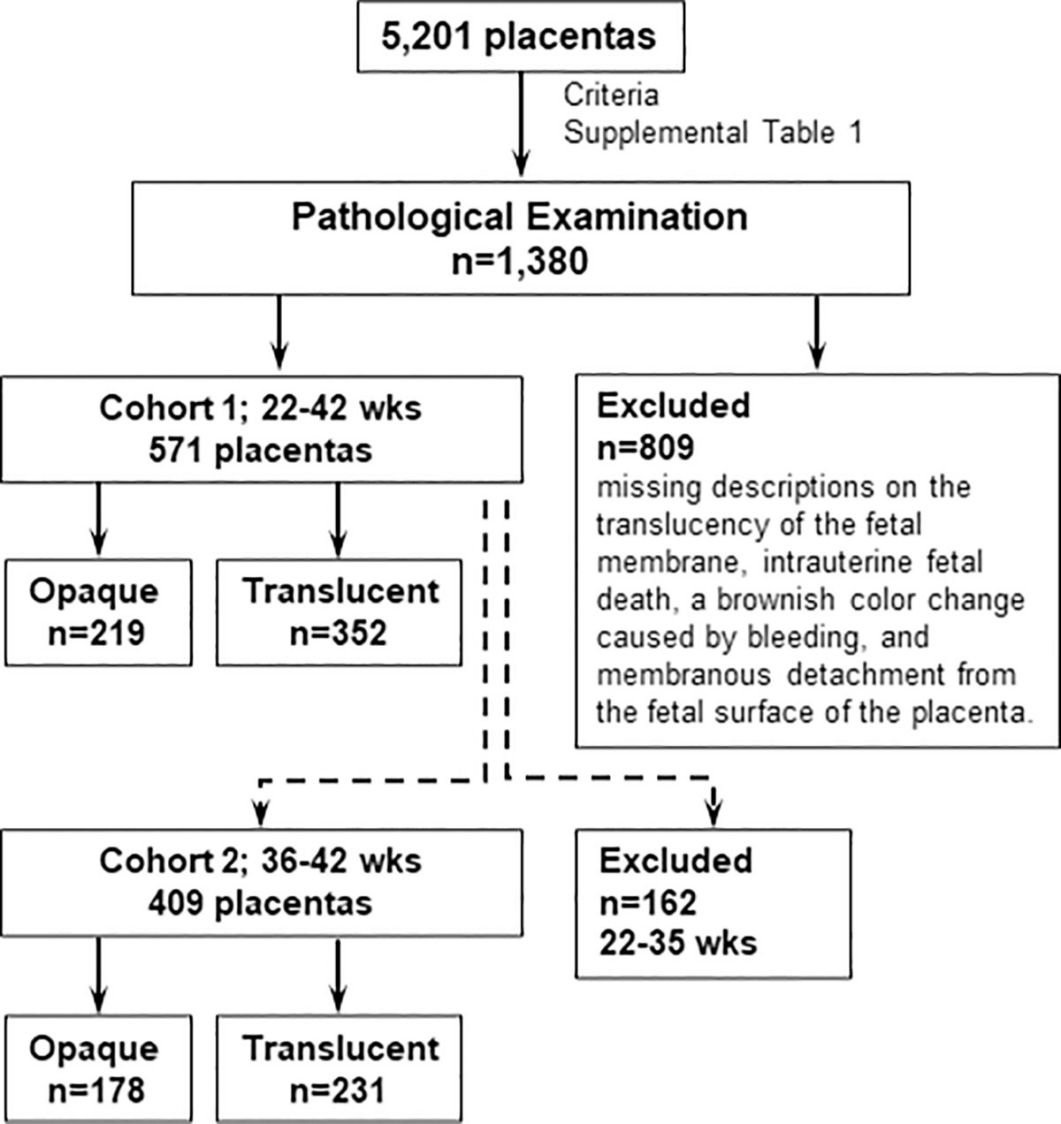
Numbers of placentas enrolled in cohorts 1 and 2.

Three researchers (Y.H., N.I., and M.M.) macroscopically evaluated 5,201 placentas delivered at Hamamatsu University Hospital between April 2010 and March 2017, among which 1380 placentas were pathologically examined according to the criteria of S1 Table as previously described [15]. Written informed consent was obtained from the participating parturient during pregnancy after a full explanation of the study. Similar procedure was applied in cases of minors. After the exclusion of cases with missing descriptions on the translucency of the fetal membrane, a brownish color change caused by bleeding, and membranous detachment from the fetal surface of the placenta, 571 placentas (22–42 wks of gestation) were retrospectively examined in the present study. Clinical backgrounds and neonatal outcomes were obtained from the clinical database. 571 placentas were obtained from mothers of 558 Japanese, 5 Brazilian, 4 Philipina, 2 Chinese, a Peruvian, and an Arab. All 571 placentas were retrospectively enrolled in Cohort 1 for comparisons between the opacity of the fetal membrane and the presence of histological chorioamnionitis (Fig 1). Among 571 placentas, 409 term placentas (36–42 wks of gestation) were separately enrolled in Cohort 2 to investigate the relationship between the opacities of fetal membranes and neonatal complications, because preterm delivery is also a considerable risk of neonatal complications (Fig 1). We adopted a criterion of 36–42 wks of gestation as term delivery according to the routine neonatal care protocol of our hospital. Since Hamamatsu University Hospital in the regional tertial perinatal center, we cannot deny the possibility the participants were not exactly representatives of the entire ordinally population.

## Methods

**Fig 2:**
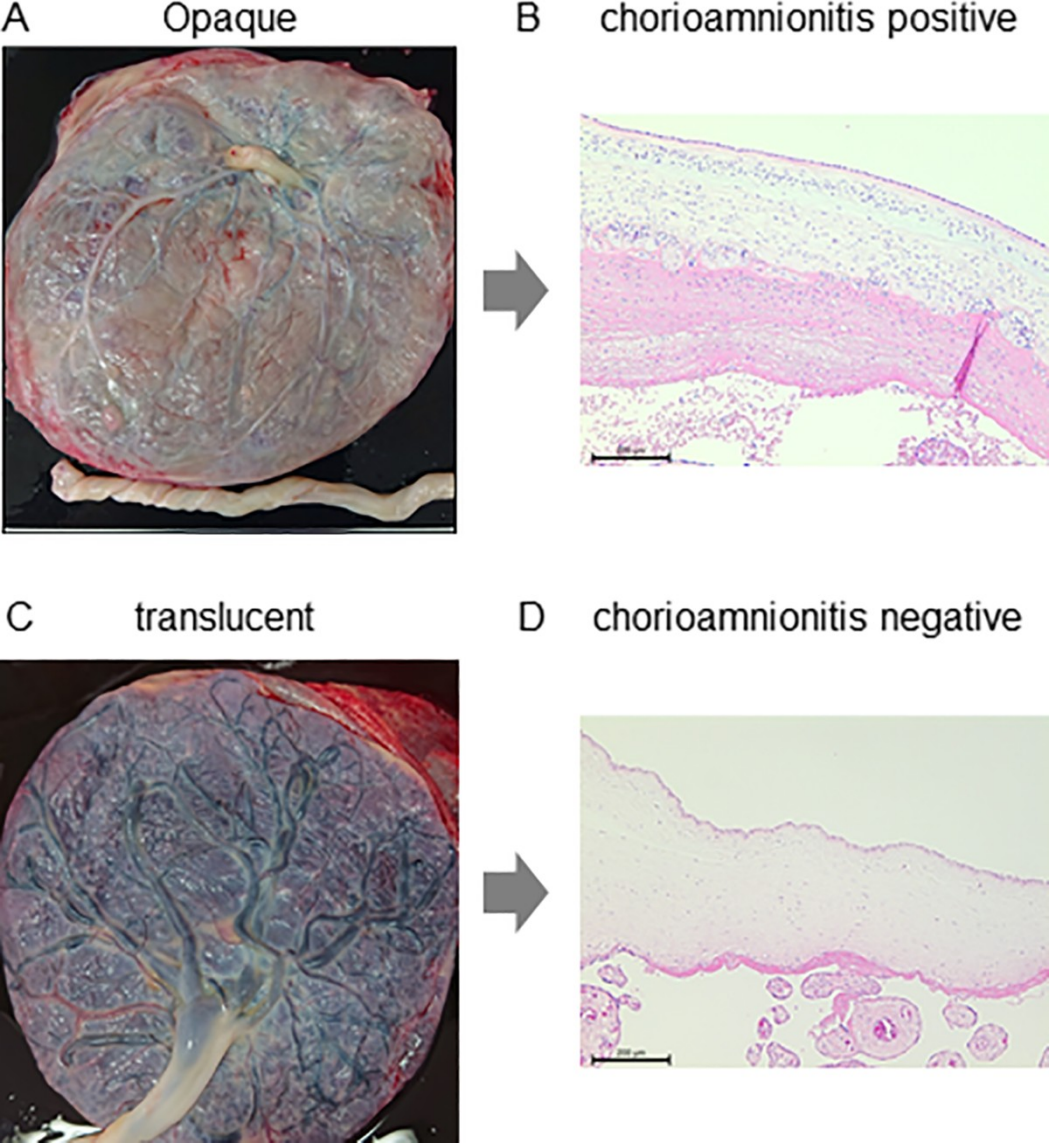
Gross appearance (A, C) and histological chorioamnionitis (B, D) of opaque (A, B) and translucent (C, D) fetal membranes. Black bars indicate 100 μm.

After delivery, placentas were stored in a refrigerator at 4°C. They were examined for the presence or absence of opacities, i.e. opaque or translucent, on the fetal surface within 24 hours. Evaluations of the color tone on the fetal surface were conducted by three researchers, and the findings obtained were decided by an open majority vote. We assessed opaque fetal membranes based on gross appearance, namely, gray to grayish-yellow discoloration and the dullness of blood vessels on the chorionic plate, which were observed through the fetal surface (Fig 2). Turbid membranes were included in the opaque group. The inter-rater reliability of the three evaluators showed 1) a percent overall agreement: 84.62%, 2) free-marginal kappa: 0.69 [0.37–1.00], and 3) fixed-marginal kappa: 0.68 [0.35–1.00] (Table 1). Placentas were then retrospectively divided into two groups with or without opacities, i.e. opaque or translucent, on the fetal membrane.

**Table 1:** Inter-rater reliability of three evaluators.

	1	2	3	4	5	6	7	8	9	10	11	12	13
**Y.H.**	S	O	S	O	O	O	S	S	S	S	S	S	S
**N.I.**	S	O	O	O	O	O	O	S	S	S	S	S	S
**M.M.**	O	O	O	O	O	O	S	S	S	S	S	S	S
**Chorioamnionitis**	**P**	**P**	**P**	**P**	**P**	**P**	**N**	**N**	**N**	**N**	**N**	**N**	**N**

Data was collected during one month, January 2017, in the research period of Cohort 1.

O; Judgment of an opaque membrane, S; Judgment of a translucent membrane, P; Positive, N; Negative.

### Preparation for the pathological examination of placentas

After they had been weighed and their gross morphology examined, whole placentas were stored in our pathological division after being vacuum-sealed in plastic packages with 10% formaldehyde, as described previously. Seven paraffin blocks were systematically obtained from each placenta for the pathological examination by systematic random sampling as previously described [15]. Two rolls of extraplacental membranes per placenta were together embedded in a block to make a single section. Each block was cut into 3-μm-thick sections and stained using hematoxylin and eosin (HE). Eight sections (seven sections from the placental parenchyma and one section from the extraplacental membrane) were analyzed per placenta.

### Histological grading of chorioamnionitis

Histological chorioamnionitis was diagnosed being based on the maternal inflammatory response of Amsterdam Placental Workshop Group Consensus Statement [16]. Four researchers (YH, NFI, MM, CY) were involved in the placental histological screening; however, the final diagnosis of chorioamnionitis was decided by a researcher CY. We retrospectively examined the relationship between the presence of an opaque fetal membrane and the incidence of chorioamnionitis.

### Assessment of early neonatal complications

We compared neonatal complications in 412 term placentas (Cohort 2) and investigated the relationship between the presence of opaque fetal membranes and the incidence of early neonatal complications. In the present study, the following complications were classified together as “chorioamnionitis-related complications”: transient tachypnea of newborns (TTN), meconium aspiration syndrome (MAS), neonatal infection, and severe neonatal asphyxia.

### Statistical analysis

Data are expressed as mean ± SDs. We used STATA, version 13.1 in statistical analyses. The two-sample Wilcoxon rank-sum test or chi-squared test was adopted for descriptive analyses where appropriate (Cohort 1; 22–42 wks of gestation) Unadjusted odds ratios (ORs) and 95% confidence intervals (CIs) for the infantile incidence of the opaque membrane to the histological incidence of chorioamnionitis as well as “chorioamnionitis-related complications” in neonates (Cohort 2; 36–42 wks of gestation) were initially compared using logistic regression analyses.

The unadjusted analysis showed a positive relationship between the incidence of an opaque membrane and the histological incidence of chorioamnionitis as well as “chorioamnionitis-related complications” in neonates among term pregnancy placentas (Cohort 2; 36–42 wks of gestation). Logistic regression analyses were subsequently performed after adjustments for maternal age, the number of births, maternal BMI, delivery mode, labor induction, and gestational age as potential confounding factors. A p-value of *<*0.05 was considered to be significant.

### Ethical considerations

The Ethics Committee of the Hamamatsu University School of Medicine approved all procedures (No. 20–102).

## Results

### Cohort 1

**Fig 3:**
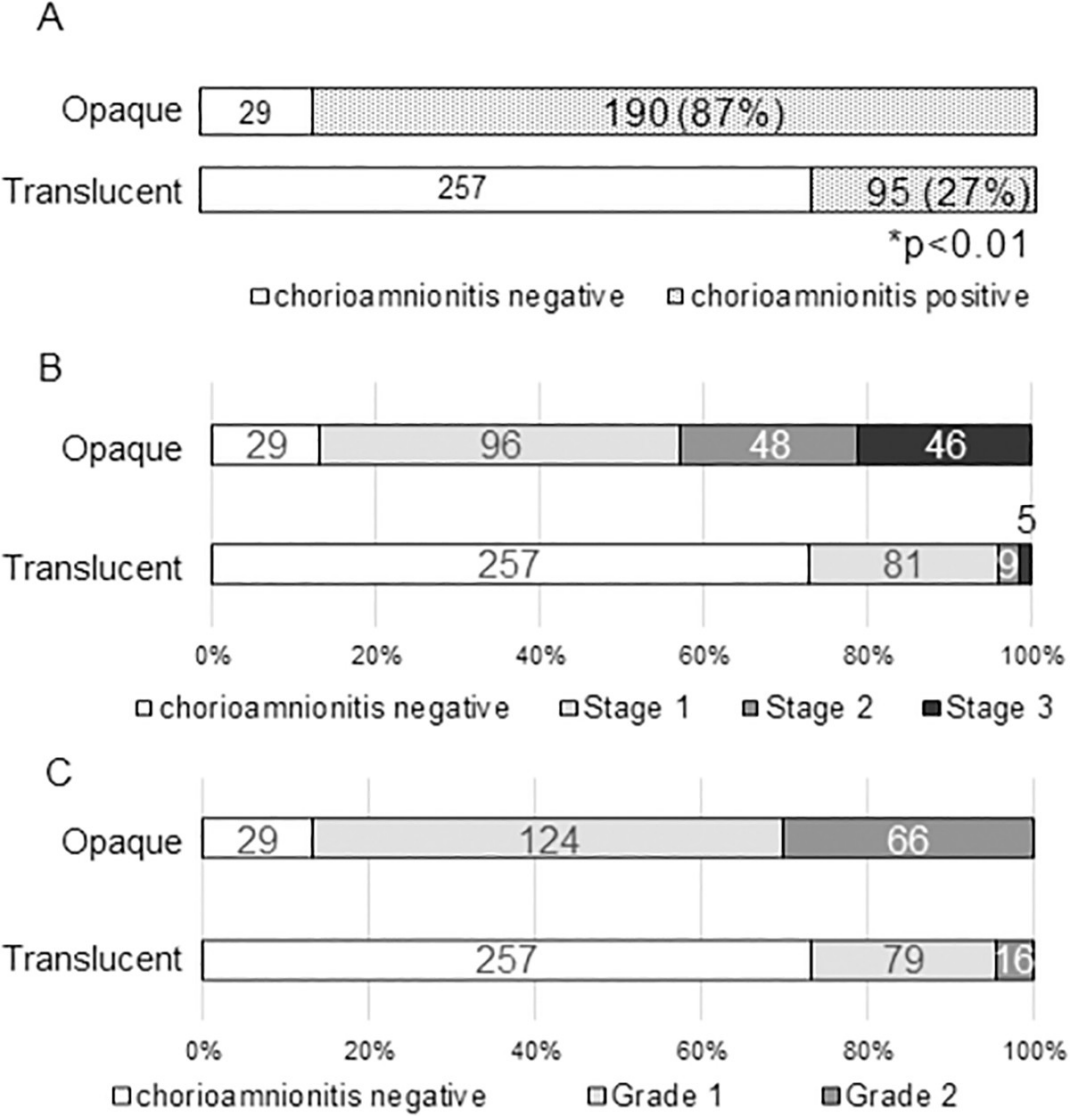
Association between Amsterdam classification of chorioamnionitis and opaque or translucent fetal membrane: entire subjects (A), stages (B), and grading (C) (cohort 1). *chi-squared test.

Among 571 placentas, opaque and translucent fetal membranes were observed in 219 and 352 cases, respectively. Table 2A summarizes the perinatal backgrounds of the participating mothers and infants in both groups. Fig 3A summarizes the number of histological identifications of chorioamnionitis in both groups. Chorioamnionitis was observed in 190 cases in the opaque group and 95 in the translucent group. A significant difference was observed between the two groups (P<0.01). Fig 3B shows the distribution of Amsterdam classification of chorioamnionitis in both groups. Stage 3 chorioamnionitis was observed in more than 90% (87/95) of cases in the opaque group. Its sensitivity and specificity were 66.7 and 89.9%, respectively, while positive and negative predictive values were 86.8 and 73.0%, respectively.

**Table 2:** Perinatal backgrounds of subjects in cohort 1 (A) and cohort 2 (B).

n = 571						Mean ± SD [range] or n			p	
						Opaque n = 219		Translucent n = 352		
**(A)**										
Maternal age (yr.)						32.1 ± 5.5 [15 – 45]		32.7 ± 5.2 [17 – 45]	0.17	
Maternal BMI (non-pregnant)						21.6 ± 4.2 [16.1–41.8]		21.4 ± 4.1 [15.2–41.3]	0.49	
Primipara						n = 147		n = 171	< 0.05	
Gestational weeks						38w1d ± 26d [23w0d - 42w1d]		36w1d ± 22d [22w6d - 41w2d]	< 0.05	
Delivery mode			Vaginal		Spontaneous	n = 126		n = 118		
					Vacuum	n = 22		n = 17		
			CS		Elective	n = 8		n = 97		
					Emergency	n = 63		n = 120		
Birth weight (g)						2788 ± 718 [576–4540]		2371 ± 659 [256–4296]	< 0.05	
Placental weight (g)						561 ± 141 [195–1000]		550 ± 200 [100–1400]	0.45	
Umbilical arterial pH						7.265 ± 0.092 [6.790–7.459]		7.297 ± 0.091 [6.597–7.555]	< 0.05	
Turbid amniotic fluid						n = 91		n = 10	< 0.05	
Neonatal complications			TTN			n = 47		n = 50		
			MAS			n = 11		n = 2		
			Severe neonatal asphyxia*			n = 7		n = 5		
			Neonatal infection			n = 5		n = 1		
			Preterm birth			n = 38		n = 121		
			Low birth weight**			n = 14		n = 50		
			Others			n = 12		n = 26		
**(B)**										
n = 409						Mean ± SD [range] or n				p
						Opaque n = 182	Translucent n = 230			
Maternal age (yr.)						32.0 ± 5.6 [15 – 45]	32.3 ± 5.4 [17 – 45]			0.55
Maternal BMI (non-pregnant)						21.4 ± 3.9 [16.1–41.9]	21.2 ± 3.9 [15.2–40.9]			0.57
Primipara						n = 131	n = 113			<0.05
Gestational weeks						39w4d ± 9d [36w1d - 42w1d]	37w6d ± 9d [36w0d - 41w2d]			<0.05
Delivery mode	Vaginal			Spontaneous		n = 106	n = 81			
				Vacuum		n = 21	n = 17			
	CS			Elective		n = 7	n = 80			
				Emergency		n = 44	n = 53			
Birth weight (g)						3009 ± 491 [1326–4540]	2665 ± 484 [1372–4296]			<0.05
Placental weight (g)						585 ± 127 [280–940]	572 ± 177 [280–1365]			0.39
Umbilical arterial pH						7.257 ± 0.083 [6.945–7.459]	7.297 ± 0.075 [6.983–7.555]			< 0.05
Turbid amniotic fluid						n = 80	n = 8			<0.05
Neonatal complications		TTN				n = 47	n = 50			
		MAS				n = 11	n = 2			
		Severe neonatal asphyxia				n = 7	n = 5			
		Neonatal infection				n = 5	n = 1			
		Low birth weight**				n = 14	n = 50			
		Others				n = 12	n = 26			

TTN: Transient tachypnea of newborns, MAS: Meconium aspiration syndrome.

*Apgar score < 4 (1 min.) **Birth weight < 2300 g.

### Cohort 2

ORs and 95% CIs were obtained between chorioamnionitis-related complications and the opaque fetal membranes of 409 placentas (36–42 weeks; Table 2B, Table 3). A correlation was found between chorioamnionitis-related complications and an opaque fetal membrane after adjustments for confounding factors, i.e. maternal age, number of births, maternal BMI, delivery mode, labor induction, and gestational age (Table 3).

**Table 3:** Odds ratios (ORs) and 95% confidence intervals (CIs) for the relationship between chorioamnionitis-related complications and an opaque fetal membrane (>36 weeks).

	Unadjusted models			Adjusted models		
	OR	95% CI.	P value	OR	95% CI	P value
Opaque fetal membrane	1.80	1.17, 2.75	< 0.01	1.82	1.07, 3.11	< 0.05
Maternal age	1.00	0.97, 1.04	0.80	1.00	0.96, 1.05	0.80
Number of births	0.98	0.75, 1.31	0.93	1.08	0.78, 1.49	0.64
Maternal BMI	1.02	0.96, 1.07	0.53	1.02	0.96, 1.08	0.51
Delivery mode	0.78	0.51, 1.20	0.25	0.95	0.58, 1.55	0.84
Labor induction	1.60	1.03, 2.49	< 0.05	1.57	0.93, 2.55	0.09
Gestational age	1.01	0.99, 1.03	0.39	0.98	0.96, 1.01	0.19

Abbreviations: OR; odds ratio, CI: confidence interval, BMI; body mass index.

Chorioamnionitis-related complications: Transient tachypnea of newborns, Meconium aspiration syndrome, Neonatal infection, Severe neonatal asphyxia|Apgar score <4 (1 min.)

## Discussion

The present study showed that the judgment of a positive opaque fetal membrane based on gross appearance correlated with histological chorioamnionitis (Cohort 1; Fig 3A). Its sensitivity and specificity were 66.7 and 89.9%, respectively, and positive and negative predictive values were 86.8 and 73.0%, respectively. 13.2% of opaque fetal membrane (29 cases) was indicative of false positive chorioamnionitis. More than half of false positive opaque fetal membrane were squamous metaplasia of epithelial cells of amniotic membrane (22/29; 75.9%). However, it is noted that approximately 90% and 84% cases of histological chorioamnionitis stage 3 and 2 were included in the opaque group, respectively (Cohort 1; Fig 3B). This is the first study to demonstrate a direct relationship between the presence of an opaque fetal membrane based on gross appearance and histologically confirmed chorioamnionitis in a large-scale cohort study, which provides scientific insights into traditional descriptions in placental textbooks [9–12].

This was a retrospective cohort study of a tertiary perinatal care center. An earlier gestational age and lower birth weight were observed in the translucent group than in the opaque group (Table 2A). We have no clear explanation of this discrepancy. Preterm delivery is a risk factor of neonatal complications. We, therefore, separately assessed the relationship with the outcomes of neonates delivered at term (Cohort 2) and found that the judgment of a positive opaque fetal membrane based on gross appearance correlated with chorioamnionitis-related complications in newborns, particularly dyspnea in term deliveries (Cohort 2; Table 3). OR was 1.80 (1.17–2.75). A correlation was observed even after adjustments for confounding factors (Cohort 2; Table 3).

Previous studies demonstrated the importance of the involvement of intrauterine inflammation later proven by the histology of chorioamnionitis in the adverse outcomes of newborns [1–4]. However, the histological detection of chorioamnionitis has not directly contributed to improvements in early neonatal care due to the delay associated with the histological diagnosis of chorioamnionitis by placental pathologists.

We herein demonstrated for the first time that the gross detection of an opaque fetal membrane was associated with the risk of chorioamnionitis-related complications in term newborns (Cohort 2; Table 3). Thus, gross detection of an opaque fetal membrane may assist in determining the risk of intrauterine infection in early neonatal care. A prospective cohort study is warranted to clarify whether the gross detection of opaque fetal membranes will contribute to better neonatal outcomes in addition to the current neonatal assessment for routine care.

There are some limitations to the present study. We excluded cases with a brownish color change caused by bleeding. A brownish change on fetal membranes suggests “diffuse chorioamniotic hemosiderosis”, which is associated with the chronic abruption-oligohydramnios sequence [17]. We examined the placental pathology after screening of the placental gross appearance and perinatal complications and did not examine uncomplicated deliveries. Since this was a retrospective cohort study at a tertiary perinatal care center, the clinical effectiveness of the gross detection of an opaque fetal membrane in early neonatal care needs to be demonstrated in unselected ordinary deliveries. The judgment of an opaque fetal membrane was performed by three obstetricians subspecializing in placental pathology. Therefore, further studies are needed to establish whether ordinary obstetricians or midwives may reliably identify the presence of an opaque fetal membrane before its introduction into routine clinical care. We did not plan the assessment of placental pathological findings other than chorioamnionitis in association with opaque fetal membrane. We did not create a standard of opaque membrane in the assessment. As for Cohort 2, we did not investigate the association between placental histological observation of fetal inflammatory response and neonatal complications, because we focused on the involvement of chorioamnionitis, maternal inflammatory response, in the neonatal complications.

## Conclusions

The present study is the first to demonstrate that the judgment of a positive opaque fetal membrane based on gross appearance correlated with histological chorioamnionitis as well as chorioamnionitis-related complications in newborns from term deliveries, and suggests the scientific relevance of a traditional routine gross inspection of the placenta soon after birth.

## Supporting information

S1 Table Criteria used in the histological examination of placentas. Intrauterine fetal death and spontaneous abortion are excluded.
(DOCX)

S1 File The minimal data set. (XLSX)
