# Supplementary material for: Gross appearance of the fetal membrane on the placental surface is associated with histological chorioamnionitis and neonatal respiratory disorders
Source: PLoS One. 2020 Nov 30;15(11):e0242579. doi: 10.1371/journal.pone.0242579 (PMC7704006; doi:10.1371/journal.pone.0242579)
Supplement: S1 Table — Intrauterine fetal death and spontaneous abortion are excluded. (DOCX) [file pone.0242579.s001.docx]

**S1 Table. Criteria used in the histological examination of placentas**

| - Neonatal asphyxia | - Clinical chorioamnionitis |
| --- | --- |
| - Neonatal dyspnea | - Transferred neonate |
| - Preterm birth (<36 weeks) | - Abnormal placenta |
| - Small for date | - Multiple gestations |
| - Maternal complications | - Other perinatal complications |

Intrauterine fetal death and spontaneous abortion are excluded.
